# Exploring body composition and physical condition profiles in relation to playing time in professional soccer: a principal components analysis and Gradient Boosting approach

**DOI:** 10.3389/fphys.2025.1659313

**Published:** 2025-10-10

**Authors:** David Ulloa-Díaz, Gabriel Fábrica-Barrios, Carlos Jorquera-Aguilera, Francisco Guede-Rojas, Jorge Pérez-Contreras, Demetrio Lozano-Jarque, Claudio Carvajal-Parodi, Luis Romero-Vera

**Affiliations:** ^1^ Department of Sports Sciences and Physical Conditioning, Universidad Católica de la Santísima Concepción, Concepción, Chile; ^2^ Unidad Académica de Biofísica, Facultad de Medicina, Universidad de la República, Montevideo, Uruguay; ^3^ Facultad de Ciencias, Escuela de Nutrición y Dietética, Universidad Mayor, Santiago, Chile; ^4^ School of Physical Therapy, Faculty of Rehabilitation Sciences, Exercise and Rehabilitation Sciences Institute, Universidad Andres Bello, Santiago, Chile; ^5^ Escuela de Ciencias del Deporte y Actividad Física, Facultad de Salud, Universidad Santo Tomás, Santiago, Chile; ^6^ Valora Research Group, Health Sciences Faculty, Universidad San Jorge, Villanueva deGállego, Spain; ^7^ Facultad de Ciencias de la rehabilitación y Calidad de vida, Escuela de kinesiología, Universidad San Sebastián, Concepción, Chile; ^8^ Facultad de Salud y Ciencias Sociales, Escuela de Ciencias de la Actividad Física, Universidad de Las Américas, Concepción, Chile

**Keywords:** soccer, body composition, physical condition, principal component, playing time

## Abstract

**Background:**

This study aimed to explore whether a predictive model based on body composition and physical condition could estimate seasonal playing time in professional soccer players.

**Methods:**

24 professional soccer players with 5–7 years of professional experience participated. Body composition and physical condition variables were assessed, and total minutes played during the season were recorded as the dependent variable. Correlations between variables were examined to reduce multicollinearity, followed by a principal component analysis (PCA) of the selected predictors. The first three components were used as inputs in a Gradient Boosting model. Model performance was evaluated using 5-fold cross-validation and leave-one-out cross-validation (LOOCV).

**Results:**

High intercorrelations among independent variables (r > 0.70) justified dimensionality reduction through PCA. The first three components explained 70% of the total variance. However, no direct correlations were observed between individual variables and minutes played, and the Gradient Boosting model did not achieve positive predictive performance under cross-validation (5-fold CV: *R* ^2^ = −0.04; LOOCV: *R* ^2^ < 0).

**Conclusion:**

In this small dataset, a multivariate approach combining PCA and Gradient Boosting did not yield predictive accuracy for playing time. Nonetheless, the PCA revealed meaningful structures in the players’ physical and body composition profiles, which may inform future research. Larger and more heterogeneous samples are required to determine whether component-based predictors can reliably estimate playing time in professional soccer.

## 1 Introduction

Soccer is a team sport with high demands and specific episodes of aerobic and/or anaerobic nature, which impose various requirements on different physiological systems (Stølen et al., 2005). The physical demands during competition and the season mean that players must develop high technical-tactical and fitness (FC) levels (Bradley et al., 2013) to execute repetitive sprints, jumps, dribbles, accelerations and decelerations with or without the ball depending on the playing position (González-Rodenas et al., 2024; García-Calvo et al., 2025). Body composition (BC) and physical condition of players are factors that influence participation in professional soccer competitions (Paoli et al., 2021; Bernal-Orozco et al., 2020).

The assessment of BF and physical condition in elite soccer players (Clemente et al., 2019; Slimani and Nikolaidis, 2019; Sebastiá-Rico et al., 2023; Cavia et al., 2019) is of great interest to medical teams, sports nutritionists, coaches and trainers (Sebastiá-Rico et al., 2023; Cavia et al., 2019). Muscle mass, fat mass in absolute and relative terms (kg and % of body weight, respectively) and the sum of six folds have been of great interest (Slimani and Nikolaidis, 2019; Sebastiá-Rico et al., 2023; Cavia et al., 2019; Sutton et al., 2009). According to Sebastiá-Rico (2023), the average muscle mass is ∼39.28 kg, which corresponds to ∼52.03% of body weight, fat mass is ∼12.48 kg, body fat percentage is ∼13.46% and skinfolds are ∑52.18–59.93 (Sebastiá-Rico et al., 2023). A lower proportion of body fat (Rienzi et al., 2000; Pedroso et al., 2024) and greater muscle mass in the lower extremities (Nikolaidis, 2014) have been positively correlated with performance in high-intensity actions, such as repeated sprints, accelerations and changes of direction, which are crucial in the most decisive phases of the game (Nikolaidis, 2014; Nikolaidis et al., 2016; Loturco et al., 2020).

The development of physical condition to withstand the demands of a season and the analysis of the short, medium and long term effects of various training systems is a constant concern of coaches and the scientific community. Determination of power and jumping (Pardos-Mainer et al., 2021; Asimakidis et al., 2024), estimation of maximal oxygen consumption (estimated VO_2max_) (Metaxas, 2021; Düking et al., 2024), ability to repeat sprints (Altmann et al., 2019; Haugen et al., 2014; Beato et al., 2021), speed and changes of direction (Fiorilli et al., 2020; Bianchi et al., 2019) are the variables that have been analyzed to the greatest extent in the performance of soccer players (Metaxas, 2021). However, there is recent interest in the study of recovery capacity, fatigue-inducing mechanisms, internal loading under the stress of competition (Mohr et al., 2005) and the study of finishing speed in different game situations (Ali, 2011) and how these components vary over the course of a season (Dolci et al., 2020).

On the other hand, the analysis of total minutes of play as an indicator of performance and competitive efficiency over the course of a season has not been sufficiently addressed in the literature and may be a crucial aspect to guide training processes (Silva, 2022), optimize performance (Silva, 2022; Della Villa et al., 2020; Arnason et al., 2004) and minimize the risk of injury (Arnason et al., 2004). As physical demands must be improved or at least maintained over the course of a season, this can be a determining factor in the number of competitive minutes over the course of a season (Dambroz et al., 2022).

Principal component analysis (PCA) is a multivariate statistical technique that allows for the identification of data patterns and has enabled the creation of profiles to assess athletic performance (Cavia et al., 2019; Sutton et al., 2009; Silva, 2022; Della Villa et al., 2020; Arnason et al., 2004; Dambroz et al., 2022). Total playing time during a season could be an indicator of performance and competitive efficiency, serving as a guide for training processes (Silva, 2022) and minimizing the risk of injuries (Arnason et al., 2004). Previous studies using PCA focused on developing training profiles (Della Villa et al., 2020), identifying athletic talent, and analyzing tactical behavior (Becerra Patiño et al., 2025) and on-field positioning (Pino-Ortega et al., 2021). However, the reviewed literature did not identify any PCA-derived patterns, based on body composition and physical fitness variables, that could establish playing time patterns for professional soccer players during a season.

However, studies of body composition and fitness variables as predictors of the minutes of play that a soccer player will have during a season have not been addressed in the literature. Therefore, the purpose of the study was to develop a predictive model capable of estimating the accumulated playing minutes during a season in professional soccer players from variables related to body composition and physical condition measured at the end of the season. The hypotheses of the study were established as a) none of the variables of body composition or physical condition, independently, explain the minutes played during a season in professional soccer players and b) the use of principal component analysis (PCA), combined with the implementation of decision trees, allows estimating the minutes played during the season and identifying sets of relevant variables.

## 2 Materials and methods

### 2.1 Study design

A four-stage analytical design was implemented; a) data processing and normalization, b) evaluation of individual relationships between independent variables and minutes played, c) dimensionality reduction through PCA and d) development and evaluation of a predictive model based on the results of the PCA. The analysis was performed considering 20 independent variables; BC (Body Mass, Height, Fat Mass, %Fat Mass, %Muscle Mass; Muscle mass% and the sum of 6 folds), PC (Jump ability for countermovement jump, Jumping Power, Linear speed 10 m, 20 m and 30 m, Speed with changes of direction of 30 m, estimated VO_2max_, Speed reached in the Yo-Yo test IR2, Fatigue index, Maximum anaerobic power, Speed of finishing in 11 m and Coordination index), the minutes of play in a season (32 matches), was considered as a dependent variable. All data collection sessions were implemented in the sports facilities during AM hours (9:00–12:00 h) on consecutive days. The study conformed to the Declaration of Helsinki. Participation was voluntary and written informed consent was obtained from participants.

### 2.2 Participants

24 male professional soccer players, with a mean age of 26.0 ± 5.61 years, body weight 76.23 ± 6.71 kg, height 176 ± 5.07 cm, with 5–7 years of sporting experience and a training frequency of seven to nine sessions per week participated in the study.

### 2.3 Procedure

**FIGURE 1:**
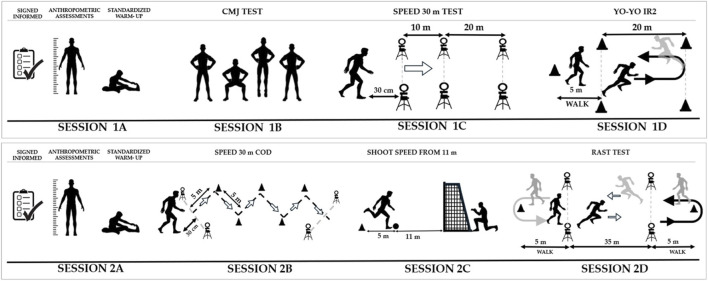
Session 1A: Warm-up and anthropometric measurements, Session 1B: Counter-movement jump, Session 1C: 30-metre speed, Session 1D: Yo-Yo IR2 assessment, Session 2A: Warm-up and anthropometric measurements, Session 2B: 30-metre speed, Session 2C: Archery shooting speed, Session 2D: Maximum aerobic power assessment (RAST).

Before each session, the players underwent a standardized 15-min warm-up which was divided into two parts. The first consisted of 7–8 min of low intensity running (heart rate less than 120 bpm) and another part specific to the predominant muscle groups in the execution of the tests. Stretching was led by the Club’s physical trainer and complemented by self-regulation exercises for each player according to their sporting experience. The players maintained their training schedules and were advised to continue with their lifestyle and diet. The 30 m speed test (10 m, 20 m and 30 m), 30m change of direction (COD 30 m), Repeated Sprint Performance Test (RAST), Yo-Yo IR2 and finishing speed tests were performed on natural grass and with competition shoes. The jumping test was performed on a regular surface and with sports shoes. The order and distribution of the evaluation is shown in Figure 1.

### 2.4 Body composition

Anthropometric assessments were taken only once before starting the first session of physical assessments (session 1). Body mass was determined with an electronic scale (Tanita TBF 300A, Tokyo, Japan) with an accuracy of 100 g. Height was measured with a portable measuring rod (Seca 213, Hamburg, Germany) with an accuracy of 1 mm. Both measurements were used for the determination of body mass index. The percent-age of Fat Mass (%FM) and the % Muscle Mass (%MM) and the sum of the 6 folds (triceps, subscapular, supraspinal, abdominal, medial thigh and calf), were calculated following the recommendations and pent compartmental protocol (Kerr, 1988), the sum of six skinfolds (triceps, subscapular, supraspinal, abdominal, medial thigh and calf) was adjusted to the Durnin and Womersley recommendations and equation (Durnin and Womersley, 1974). All measurements conformed to the recommendations of the International Society for the Advancement of Kinanthropometry (ISAK) (Marfell-Jones et al., 2006) and were taken by trained personnel with 5 years of experience and ISAK certification.

### 2.5 Countermovement jump

2.2 After the standardized warm-up all participants performed a maximal jumping test in a CMJ exercise following the instructions reported by Bosco (Bosco et al., 1983). Jump height was calculated with an Optojump Next infrared recording system (Microgate, 2023; Bolzano, Italy) and jump power was calculated according to the following equation:

$$P=2.214\;x\;body\;weight\;x\;\sqrt{jump}\;height$$

After a series of preparatory countermovement jumps, subjects initiated the jump from a bipedal position with knees extended, descended to 90° knee flexion and immediately performed an explosive concentric action of the lower limb extensor musculature to reach maximum height. The jump height was calculated from the flight time, the highest of three attempts was recorded.

### 2.6 Linear speed of 30 m

Linear speed was assessed in a 30 m sprint, with 10 m, 20 m and 30 m recordings. The test started from a stationary position 30 cm from the first photocell. The sprint time was recorded using the Witty-Gate photocell system (Microgate, Bolzano 2023; Italy). Three attempts were made at the test, separated by a 2-min rest period, and the best score was recorded.

### 2.7 VO_2max_ estimation (Yo-Yo IR2)

After 10 min of the speed test and to estimate VO_2max_, participants performed the Yo-Yo Intermittent Recovery Test level 2 (Bangsbo et al., 1991; Krustrup et al., 2003). The test was conducted following the recommendations of Bangsbo (Bangsbo et al., 2008). An audible signal was played from an-iPhone handheld device (Apple Inc., Cupertino, CA) connected by Bluetooth to a player, which was placed perpendicular to the 20-m running lanes. Between each out and back run (40 m), participants had a 10 s rest period of active recovery, where they had to move to a signal 5 m away before returning to the starting line. The test was considered completed when participants withdrew voluntarily or at the instruction of the assessors. The final distance and speed achieved were recorded for analysis. Estimated maximal oxygen consumption was calculated using the equation:

$$VO2max\;\left(ml\cdot \;\min-1\cdot \;kg-1\right)=IR2\;distance\;\left(m\right)\;\cdot \;0,0136+45,3$$

### 2.8 Speed with changes (COD 30m)

In session 2 and after the standardized warm-up, the speed was determined in a 30 m sprint with 90° changes of direction every 5 m. The test started from a stationary position 30 cm from the first photocell and the best of three attempts was recorded.

### 2.9 Shoot speed from 11m

The shoot speed was recorded for the dominant leg and three others with the non-dominant leg from a distance of 11 m to the monitoring system. Speed was recorded with a Stalker Sport2 radar system (United States, 2024), which was positioned behind the 11 m line. The highest speed was recorded for both segments. The Shoot Coordination Index (SCI) was determined as the percentage difference between the ball striking speed of the dominant and non-dominant leg.

### 2.10 Repeated sprint performance (RAST)

The RAST test was used to verify repetitive sprint performance (De Andrade et al., 2016). All participants performed 6 sprints of 35 m at maximum speed interspersed by 10 s of active recovery. The test was controlled by the photocell system. Three experienced testers controlled the test, one of the testers was positioned at the start and one at the end of the 35 m track to control the recovery time (10 s) and the good execution of the test. The third evaluator recorded the time of each sprint. Power was assumed to be the product of strength and speed for each effort (Power = speed - strength). The speed for the 6 efforts was used to establish the fatigue index (FRI) and the power of each sprint was used to establish the maximal anaerobic power. Table 1 shows the means, standard deviations, maximum and minimum values of the variables of body composition, physical condition and minutes of play.

**TABLE 1:** Body composition and physical condition variables analyzed.

	Mean	SD	Max	Min
Body Mass (kg)	76.23	6.71	90.20	66.10
Size (cm)	176.20	5.07	184.30	168.10
%FM	20.67	2.18	25.32	16.96
%MM	51.43	1.96	55.08	47.91
Σ 6 Folds (mm)	50.42	9.65	66.70	30.50
CMJ (cm)	40.55	4.48	48.20	30.40
Jumping Power (W)	1,073.47	111.98	1,285.48	806.89
Distance Yo-Yo (m)	723.33	175.32	1,160	480
Speed Yo-Yo (m·s-1)	5.77	0.15	6.16	5.58
VO2max E (mL·kg-1·min-1)	55.14	2.38	61.08	51.83
Speed 10m (m·s-1)	5.79	0.21	6.21	5.43
Speed 20m (m·s-1)	8.03	0.36	8.55	7.27
Speed 30m (m·s-1)	7.12	0.28	7.58	6.59
Speed COD (m·s-1)	3.22	0.18	3.46	2.87
% Change	−54.72	2.05	−59.30	−51.08
Fatigue Index (%)	14.51	7.71	37.55	6.60
MAEP (W)	775.12	121.99	1,037.43	588.52
Dominant shoot (m·s-1)	108.42	4.67	114.50	96.20
Non-dominant shoot (m·s-1)	96.13	8.14	104.40	92.00
Coordination index (%)	−11.30	7.07	−34.85	−2.14
Minutes of play (min)	815.91	814.84	2,275	0

Abbreviations: %FM, percentage fat mass; %MM, percentage muscle mass; MM, millimeters; CMJ, countermovement jump; VO2max E, estimated maximum oxygen consumption; COD, change of direction; MAEP, maximum anaerobic power.

### 2.11 Ethical approval

The Ethical approval was obtained from the Universidad Católica de la Santísima Concepción, (registration number: 01/2024). Participants provided informed consent, which included comprehensive details about the research, associated risks, potential benefits, confidentiality measures, and participant rights. The study strictly adhered to the ethical principles outlined in the Declaration of Helsinki, ensuring the protection of participants’ rights and wellbeing throughout the design, procedures, and confidentiality measures. All stages of this study complied with the Helsinki guidelines for human research and met the current ethical standards in Sport and Exercise Science.

### 2.12 Statistics

Descriptive data are summarized by means and standard deviations. An initial reduction in the number of independent variables was carried out, eliminating those highly correlated with each other to avoid redundancies. Prior to inferential analysis both the independent variables and the dependent variable (minutes played) were normalized using the Min-Max scaling method, to transform the values to a range between 0 and 1, maintaining the original relative distribution and ensuring comparability between different units of measurement. The distribution of each variable was assessed using the Shapiro-Wilk test (p ≤ 0.05). To identify individual associations between each independent variable and minutes played, Pearson’s correlation coefficients were calculated. The magnitude of the correlation was interpreted as low r < 0.30; moderate: 0.30–0.70 and high: ≥0.70 (Field, 2013), in all cases a confidence level of 95% was considered. Subsequently, a Principal Component Analysis (PCA) was conducted on the seven normalized independent variables. Component retention was guided by three complementary criteria: inspection of the scree plot, the Kaiser criterion (eigenvalues >1), and cumulative variance explained. Both unrotated and Varimax-rotated solutions were examined; however, the unrotated solution was retained to preserve orthogonality and the ordered maximization of explained variance, which were required for subsequent predictive modelling. Loadings ≥ |0.30| were considered meaningful contributors (Field, 2013). The first three components were then used as predictors in a Gradient Boosting model (Friedman, 2001) to estimate minutes of play. Model performance was assessed using 5-fold cross-validation with shuffling, complemented by leave-one-out cross-validation (LOOCV) as a sensitivity analysis. A leakage-free pipeline was implemented, combining scaling, PCA, and Gradient Boosting. To further limit overfitting, hyperparameters (learning rate, number of estimators, maximum depth, and subsampling rate) were tuned via grid search within the cross-validation framework. Performance was evaluated on out-of-fold predictions using the coefficient of determination (*R* ^2^), mean squared error (MSE), and mean absolute error (MAE). All analyses were performed in Python 3.9.

## 3 Results

**FIGURE 2:**
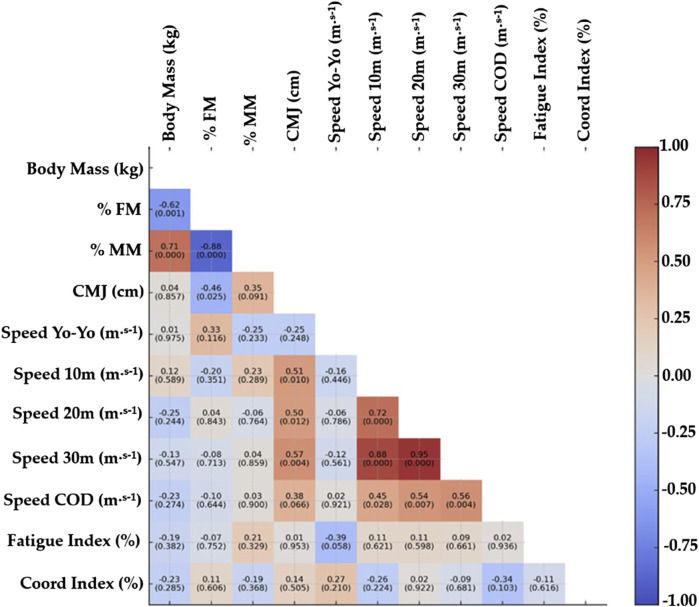
Correlation matrix of study variables. Abbreviations: %FM: percentage fat mass, %MM: percentage muscle mass, MM: millimetres, CMJ: countermovement jump, Speed Yo-Yo IR2: Speed test Yo-Yo intermittent resistance level 2, Speed 10 m: Speed of 10 m, Speed COD 30 m: Speed change of direction 30 m.

Figure 2 shows the results of the correlation analysis between the independent variables. High correlations are observed between BM, %FM and %MM, 10 m, 20 m and 30 m speed and 30 m speed with change of direction.

Correlational analysis of each of the remaining 7 independent variables and minutes of play showed no strong association. The correlation coefficients obtained were: %MM (r = 0.47), Fatigue Index (r = 0.22), Coordination Index (r = −0.09), CMJ (r = −0.10), Speed 10 m (r = 0.28), Speed Yo-Yo (r = 0.28) speed COD (r = −0.39).

The PCA indicated that the first three components explained 34.2%, 21.4% and 14.3% of the variance, respectively, for a cumulative total of 70%. According to both the Kaiser criterion and the scree plot, the retention of three components was justified. Although a Varimax rotation was tested, it resulted in more diffuse loadings and did not improve interpretability. In addition, since rotation disrupts the ordered maximization of variance that is essential for predictive modelling, the unrotated solution was selected for the final analysis. The interpretation of the principal components was based on selecting, for each variable, the two components with the highest loadings, and then identifying the two or three most representative variables per component. This approach was subjective but commonly used, as no standard criterion is universally established. In the selection of variables, although we had set a threshold load of 0.30 for component interpretation (Field, 2013), the values were within more stringent thresholds (e.g., >0.40–0.70) applied in sports science studies (Rojas-Valverde et al., 2020). The factor loadings matrix for each variable in the first three components is presented in Table 2.

**TABLE 2:** Matrix of factor loadings for each variable in the first three components.

	PC1	PC2	PC3
%MM	−0.34	−0.42	−0.43
CMJ (cm)	**−0.44**	0.04	−0.51
Speed Yo-Yo IR2 (m·s^-1^)	0.27	**0.54**	−0.25
Speed 10m (m·s^-1^)	**−0.52**	0.15	−0.08
Speed COD 30m (m·s^-1^)	**−0.52**	**0.54**	0.33
Fatigue index (%)	−0.17	**−0.45**	0.39
Coordination index (%)	0.21	0.03	−0.49

Abbreviations: PC, principal component; %MM, percentage muscle mass; CMJ, countermovement jump; Speed Yo-Yo IR2, Speed test Yo-Yo intermittent resistance level 2; Speed 10m, Speed of 10 m; Speed COD, 30m, Speed change of direction 30 m. Values highlighted in bold indicate the variables selected for component interpretation. Values in bold: statistical significance.

Cross-validated performance was very limited. With 5-fold CV, Gradient Boosting on the three PCA components did not achieve positive predictive performance (*R* ^2^ ≤ 0 in CV/LOOCV). These results contrast with the higher *R* ^2^ = 0.75 observed under a single 80/20 split, indicating that the latter likely overestimated predictive accuracy due to sampling variability and the very small test set (n = 5). The updated cross-validated metrics (*R* ^2^, MSE, MAE) are presented in Table 3. Overall, these findings demonstrate that the predictive model should be regarded as exploratory, with limited generalizability in the current dataset.

**TABLE 3:** Cross-validated performance metrics.

Validation strategy	*R* ^2^	MSE	MAE
5-fold CV (default hyperparameters)	−0.204	791,094	692.9
5-fold CV (tuned hyperparameters, nested)	−0.041	654,998	667.1
LOOCV (default hyperparameters)	−0.056	703,728	678.9

Abbreviations: *R* ^2^, coefficient of determination; MSE, mean square error; MAE, mean absolute error.

## 4 Discussion

The purpose of the study was to determine the relationship between variables of body composition, physical condition and their relationship with minutes of play in professional soccer players in a regular season of 32 games. The following hypotheses were established: a) none of the body composition or physical condition variables, independently, explain the minutes played during a season in professional soccer players and b) the use of principal components analysis, combined with the implementation of decision trees, allows estimating minutes played during the season and identifying sets of variables that are relevant. Although the first hypothesis is fulfilled, the second is not completely, since with this data set Gradient Boosting approach did not achieve positive predictive performance. However, the approach allowed to identify relevant variables and test the methodological feasibility.

Significant correlations (r > 0.7; p < 0.05) were found between the BC variables of BM, %FM and %MM and the PC variables of 10m, 20m and 30m speed and COD 30m. Previous studies analyzing the relationship between BC and different physical performance variables in elite soccer players have identified significant associations that reinforce the importance of a BC profile optimized for the demands of the game (García-Calvo et al., 2025; Paoli et al., 2021; Bernal-Orozco et al., 2020; Clemente et al., 2019; Slimani and Nikolaidis, 2019; Sebastiá-Rico et al., 2023; Cavia et al., 2019; Sutton et al., 2009; Rienzi et al., 2000; Pedroso et al., 2024; Nikolaidis, 2014). A lower proportion of body fat (%BF) tends to correlate positively with performance in high-intensity actions, such as repeated sprints, accelerations and changes of direction, which are essential in the most decisive phases of the game (Dalen et al., 2016), which is consistent with our results. This could be explained by the increase in energy availability and mechanical efficiency, which would be favored by a reduced fat mass, by reducing the inertial load during movements (Pedroso et al., 2024; Nikolaidis, 2014). In contrast, a high muscle mass in the lower limbs is a determining factor in performance in explosive strength tests, such as the CMJ and short sprints associated with real game situations (Pedroso et al., 2024; Nikolaidis, 2014; Nikolaidis et al., 2016). This finding highlights the importance of developing lean mass in key areas for power generation and for the performance of technical gestures at high intensity (Reilly et al., 2000). The results suggest that fat and muscle mass in relative terms have a significant influence on performance, justifying their inclusion in the evaluation and training planning processes in elite soccer (Oliver et al., 2024). However, these results also show that the use of these variables may be redundant, which should be considered when designing some statistical analyses.

On the other hand, the results of the individual correlations between the remaining independent variables and minutes played (all r < 0.70, p > 0.05), showed that none of these variables, on their own, have a direct explanatory weight on competitive participation throughout the season. This finding supports our first hypothesis and justifies the use of a multivariate approach such as PCA, given that the relationships could be collinear, non-linear or interdependent. Although the absence of strong individual correlations with minutes played might appear predictable, the use of PCA provided added value beyond this initial observation. Specifically, PCA reduced the redundancy inherent in highly collinear variables, allowing for the extraction of latent dimensions that summarized complex performance profiles. These components captured meaningful constructs such as explosive power, anaerobic endurance, and agility, which could not be identified through single-variable analyses. This multivariate perspective is consistent with recommendations in sports science to apply PCA when dealing with interdependent physiological and anthropometric data (Pino-Ortega et al., 2021; Rojas-Valverde et al., 2020). Thus, PCA contributed to a more nuanced understanding of how physical and body composition variables cluster, offering a framework that can be extended to larger and more diverse samples in future research. In practical terms, the application of PCA can help reduce the number of evaluations to those that truly contain relevant information regarding a given factor.

In our study, the first three components extracted by PCA explained 70% of the total variance of the independent variables. The relative analysis of the weights of the variables in the components obtained in the PC1 shows that Speed 10m (−0.52), COD 30 m (−0.52) and CMJ (−0.44) are relevant. Given that these are negative values, this component can be associated with low values of speed and power (Dambroz et al., 2022; Taylor et al., 2022). PC2 seems to capture endurance and agility in short movements, given that Yo-Yo speed (0.54) and COD 30 m (0.54) are the main related variables. Here positive values indicate that players with higher values in that variable will have higher scores in that component (Russell et al., 2011). In addition, this component includes the fatigue index with a negative value (−0.45). Since a higher index reflects less anaerobic endurance (Riley et al., 2024), its value within the component indicates that players with higher anaerobic endurance will have higher scores in PC2. As for PC3, its association with CMJ (−0.509), %MM (−0.417), and the coordination Index (−0.49), suggest that it captures power in both physical and technical actions, in this case its interpretation goes in the same direction as PC1.

By incorporating the three main components into a Gradient Boosting model, no positive predictive performance was achieved under cross-validation, indicating that the multicomponent approach could not reliably estimate minutes played in this small dataset. Confidence intervals were not reported due to the small sample size, which would render such estimates unstable (Ghasemzadeh et al., 2024). Instead, robustness was assessed through cross-validation procedures (5-fold CV and LOOCV), providing a more reliable indication of model generalizability. Indeed, studies have shown that statistical confidence can be up to four times higher when using nested cross-validation compared to simple methods such as hold-out (Ghasemzadeh et al., 2024). The initial 80/20 split suggested an apparent predictive signal, but this was likely due to overestimation arising from sampling variability and the very small test set. Indeed, recent work has shown that models trained on small datasets systematically overestimate predictive performance, and that substantially larger samples are required to obtain stable estimates of generalizability (Zantvoort et al., 2024). Consequently, the model should be regarded as exploratory, highlighting the need for larger and more heterogeneous samples to evaluate whether component-based predictors can meaningfully account for seasonal playing time. In the initial 80/20 split analysis, PC2 showed the highest relative influence, followed by PC1 and PC3. However, since cross-validation revealed no predictive accuracy, these relative weights should be regarded solely as descriptive of how the model fitted this specific split, and not as reliable indicators of component importance.

## 5 Conclusion

The use of a multivariate approach allowed us to identify combinations of physical and body composition variables that, when integrated into principal components, summarized relevant aspects of players’ profiles. Although Gradient Boosting applied to these components did not yield reliable predictive accuracy for competitive playing time in this small dataset, the analysis highlighted dimensions such as anaerobic endurance, high-intensity aerobic capacity, change-of-direction speed, and muscle power as important elements within the component structure. These results emphasize the potential of tools such as PCA and Gradient Boosting for exploring complex relationships in sports data, but also demonstrate the need for larger and more heterogeneous samples to evaluate their predictive value. Rather than providing a tool to anticipate season participation, the present findings should be regarded as exploratory evidence that may guide future investigations into training, injury prevention, and performance management. Although there may be other aspects that can influence the minutes of the season, such as technical and tactical decisions and the state of health of the athlete.

### 5.1 Limitations

This study was conducted with a small sample of 24 players from a single professional club. Therefore, the results cannot be generalized to all professional football players and should be regarded as exploratory findings specific to this cohort. The limited sample size also restricts the statistical power of the analyses and increases the risk of overfitting in the predictive models, even when cross-validation procedures were applied. Moreover, the use of total minutes played across the entire season does not capture temporal fluctuations related to player form, injuries, or coaching decisions, which likely influenced match participation. Finally, the interpretation of principal components and their integration into the Gradient Boosting model should be considered preliminary, as larger and more heterogeneous datasets will be required to confirm and extend these observations.

### 5.2 Applicability and future research

Future studies should consider integrating technical–tactical indicators into PCA, as these may represent additional key factors influencing the minutes of play accumulated over a season. It is also important to include variables reflecting internal and external load throughout the competitive calendar, as well as information on time lost due to injuries or suspensions, since these aspects directly affect player availability. While the present study did not achieve predictive accuracy, the use of PCA and Gradient Boosting illustrates a methodological pathway for exploring complex multivariate relationships in professional football. Larger and more heterogeneous samples are required to test whether component-based profiles combining body composition, physical condition, tactical indicators, and contextual factors can meaningfully predict playing time. If validated in future research, such models could provide medical, performance, and coaching staff with tools to support injury prevention, training feedback, recruitment, and player selection.

## Data Availability

The original contributions presented in the study are included in the article/Supplementary Material, further inquiries can be directed to the corresponding author.
